# Anthropometric Characteristics, Neuromuscular Performance and Cardiorespiratory Fitness of Chilean Youth Soccer Players: A Cross-Sectional Study

**DOI:** 10.3390/jfmk11030363

**Published:** 2026-09-09

**Authors:** Gonzalo Arias-Álvarez, Hellen Belmar-Arriagada, Waldo Osorio-Torres, Andrea González-Escobar, Víctor Arévalo-Parra, Edzon Riquelme-Yáñez, Mario Muñoz-Bustos, Oscar Rincón-León

**Affiliations:** 1 Escuela de Kinesiología, Facultad de Salud y Ciencias Sociales, Universidad de las Américas, Concepción 4090940, Chile; 2 Escuela de Kinesiología, Facultad de Ciencias de la Rehabilitación y Calidad de Vida, Universidad San Sebastián, Concepción 4080871, Chile; wosoriot@docente.uss.cl (W.O.-T.); agonzaleze@docente.uss.cl (A.G.-E.); victorarevaloparra@gmail.com (V.A.-P.); 3 Escuela de Obstetricia, Facultad de Ciencias para el Cuidado de la Salud, Universidad San Sebastián, Concepción 4080871, Chile; edzon.riquelme@uss.cl; 4 Departamento de Kinesiología, Facultad de Medicina, Universidad de Concepción, Concepción 4030000, Chile; marmunozb@udec.cl; 5 Facultad de Ciencias Económicas y Administrativas, Departamento de Economía, Pontificia Universidad Javeriana, Bogotá 110231, Colombia; osrincon@javeriana.edu.co

**Keywords:** adolescent athletes, quadriceps strength, countermovement jump, aerobic fitness, physical profiling, performance monitoring, youth soccer

## Abstract

**Background and Objectives**: Comprehensive physical profiling is important for monitoring athletic development in youth soccer players (YSP), yet integrated data from competitive South American players remain limited. This study aimed to characterize the anthropometric profile, neuromuscular performance, and cardiorespiratory fitness of competitive Chilean YSP and explore associations among these components. **Methods**: This cross-sectional study included 24 male YSP from a Chilean professional club academy. Participants underwent anthropometric assessment, bilateral quadriceps isometric strength testing, vertical jump testing (Squat Jump [SJ], Countermovement Jump [CMJ], and Abalakov Jump [ABK]), and maximal cardiopulmonary exercise testing. The primary analysis was an integrated characterization of anthropometric, neuromuscular performance, and cardiorespiratory fitness. Secondary exploratory analyses included correlations with false discovery rate adjustment and multiple linear regression. **Results**: Participants had a median age of 13.5 years (IQR: 13.0–14.0) and a BMI of 21.1 ± 1.6 kg·m^−2^. Mean bilateral quadriceps strength was 406.1 ± 70.0 N, while mean jump heights were 24.6 ± 4.2 cm for SJ, 28.4 ± 3.7 cm for CMJ, and 33.4 ± 5.3 cm for ABK. The average relative VO_2_peak was 55.0 ± 4.7 mL·kg^−1^·min^−1^, while absolute VO_2_peak was slightly higher than predicted. Bilateral quadriceps strength was positively associated with all three jumps before FDR adjustment; after FDR correction, the associations with CMJ and ABK remained statistically significant, whereas the association with SJ did not. Relative VO_2_peak was not associated with CMJ performance. **Conclusions**: This study provides an integrated characterization of physical performance within this cohort of youth soccer players. Quadriceps strength was associated with vertical jump performance, whereas relative VO_2_peak was not associated with CMJ, supporting assessment of neuromuscular and cardiorespiratory capacities.

## 1. Introduction

Youth soccer players (YSP) are exposed to progressively greater physical demands as training volume, competitive level, and tactical complexity increase throughout adolescence [1]. During training and match play, YSP are required to repeatedly perform explosive actions such as accelerations, decelerations, changes of direction, jumps, and sprints while maintaining the ability to sustain prolonged periods of moderate-to high-intensity activity. Consequently, successful performance depends on the interaction of multiple physical capacities rather than on a single physiological attribute [2,3].

Given these demands, physical assessment has become an essential component of player development in youth soccer [4,5]. Rather than relying on isolated performance tests, current evaluation strategies commonly employ multidimensional assessment batteries that integrate anthropometric characteristics, neuromuscular performance, and cardiorespiratory fitness [6,7]. These evaluations are used to monitor training adaptations, identify individual strengths and weaknesses, guide exercise prescription, and support long-term athlete development. Assessing these domains within the same athlete provides a more comprehensive understanding of physical performance than evaluating each domain in isolation [8].

Each domain contributes complementary information to the overall physical profile of the athlete. Anthropometric assessment describes body size and composition during growth and maturation, providing important context for interpreting physical performance [9,10,11]. Neuromuscular testing quantifies lower-limb force production and explosive performance, capacities that underpin actions such as jumping, sprinting, and rapid changes of direction [7]. In particular, quadriceps muscle strength and vertical jump testing are widely used in the literature. Among vertical jumps, the countermovement jump (CMJ) is the most widely used and ecologically valid indicator of lower-limb neuromuscular performance in soccer players, reflecting the contribution of the stretch–shortening cycle during explosive movements [12]. Cardiorespiratory fitness, commonly assessed through peak oxygen uptake (VO_2_peak), reflects the ability to sustain repeated high-intensity efforts and recover efficiently between them during training and competition [13,14]. Together, these measures allow a multidimensional characterization of the physical attributes that support soccer performance during adolescence [15].

Previous studies have investigated anthropometric characteristics, muscular strength, vertical jump performance, and cardiorespiratory fitness in YSP across different age groups, competitive levels, and playing positions [16,17]. Collectively, these investigations have substantially advanced the understanding of physical development in YSP [18]. However, most have focused on individual physical domains or a limited number of performance outcomes. At the South American level, most of the evidence comes from Brazil, where the sport is highly developed. In Chile, the studies by Martínez and Villaseca [16,19] provide some reference values, but these relate to physical profiles associated primarily with anthropometric variables and physical performance measured through field tests. This opens up the possibility of expanding the local evidence base to include a more comprehensive characterization of physical performance. Consequently, evidence integrating anthropometric, neuromuscular, and cardiorespiratory performance within the same cohort using a standardized assessment battery remains limited, particularly in South American youth soccer populations. Furthermore, the influence of the context in which a YSP takes place is widely recognized; this context undoubtedly varies across different local competitions, highlighting the complexity of generalizing sports performance data [17].

A multidimensional characterization of physical performance may improve the interpretation of routine assessments by providing a more comprehensive description of the athlete’s physical profile [20]. Because anthropometric characteristics, neuromuscular performance, and cardiorespiratory fitness represent complementary domains of physical performance, examining them together may provide information that is not captured when each domain is evaluated separately. Furthermore, exploring the relationships among these domains may contribute to a better understanding of how physical attributes interact during adolescence, a period characterized by non-linear and asynchronous changes in body size, strength, and aerobic capacity associated with biological maturation [21,22,23].

Therefore, the aim of this study was to characterize the anthropometric characteristics, neuromuscular performance, and cardiorespiratory fitness of competitive Chilean YSP using an integrated assessment battery. As secondary exploratory analyses, we examined the associations among these physical domains and explored the associations of selected neuromuscular and cardiorespiratory variables with countermovement jump performance, a widely used indicator of lower-limb explosive performance.

## 2. Materials and Methods

### 2.1. Study Design

This observational cross-sectional study was conducted at the Kinesiology Center of Universidad San Sebastián (Concepción, Chile) between October and November 2025. The study was designed and reported in accordance with the Strengthening the Reporting of Observational Studies in Epidemiology (STROBE) statement for observational studies [24].

The study protocol was approved by the Scientific Ethics Committee of Universidad San Sebastián (Project No. 213-24; approval date: 28 January 2025) and was conducted in accordance with the ethical principles of the Declaration of Helsinki [25]. Because all participants were younger than 18 years, written informed consent was obtained from their parents or legal guardians, and written informed assent was obtained from each participant before enrollment and data collection.

### 2.2. Participants and Eligibility Criteria

Players were recruited from the youth academy of Deportes Concepción (Concepción, Chile) using a convenience sampling approach. The 30 players invited to participate represented all eligible players available from the club’s youth development program during the recruitment period. Therefore, no a priori sample-size calculation was performed. Of these, 24 completed all assessments and were included in the final analysis. Six players did not complete the assessment protocol because of acute respiratory illness (*n* = 3) or musculoskeletal injury (*n* = 3) and were therefore not included in the final analysis. Throughout the study period, players were regularly involved in structured soccer training and official competition according to their respective age categories. Players completed four supervised field-based training sessions per week (Tuesday to Friday), totaling approximately 5 h 45 min of structured soccer training, in addition to one official match per week during the competitive season. As part of their regular in-season conditioning program, players also completed one supervised resistance-training session (~30 min) per week. At the time of testing, all included players were free from musculoskeletal conditions or other medical issues that could limit maximal physical performance.

Eligibility criteria. Participants were eligible if they were male soccer players younger than 18 years, officially registered with Deportes Concepción, and eligible for regular training and competition within the club’s youth development program. Players were excluded if they had sustained a lower-limb musculoskeletal injury within the previous three months, reported pain during testing, had a diagnosed condition associated with functional limitation, presented any medical contraindication to maximal exercise testing, or were unable to complete the assessment protocol.

Before enrollment, all players and their parents or legal guardians received written and verbal information regarding the study procedures. Participation was voluntary, and players were free to withdraw from the study at any time without consequences.

### 2.3. Assessment Procedures and Study Variables

Prior to the assessment, participants were instructed to wear appropriate sports clothing and athletic footwear, consume their usual breakfast at least 1 h before testing, maintain their usual hydration status, avoid vigorous physical activity and caffeinated or energy-containing beverages for at least 24 h before testing, and attend the evaluation accompanied by a parent or legal guardian. All assessments were completed during a single testing session by trained evaluators following standardized testing procedures. Before testing, participants completed an administrative and clinical screening station, during which eligibility criteria were verified, written informed consent and assent were obtained, and a brief clinical history was recorded. The evaluation was organized into dedicated assessment stations, with each station supervised by the same evaluator throughout the study to ensure procedural consistency. The assessment protocol was performed in the following order: clinical history, anthropometric assessment, quadriceps isometric strength, vertical jump performance, and cardiopulmonary exercise testing. To minimize the potential effects of fatigue, participants were provided with a standardized 15-min seated recovery period between the quadriceps strength and vertical jump assessments and again before cardiopulmonary exercise testing. All assessments were conducted during the final stage of the competitive season in the same gymnasium under standardized environmental conditions between 09:00 and 13:00 h.

#### 2.3.1. Anthropometric Measurements

Standing height and body mass were assessed using standardized procedures. Standing height was measured to the nearest 0.1 cm using a portable stadiometer (Seca 213, Seca GmbH, Hamburg, Germany), with participants standing barefoot in an upright position, heels together, and the head positioned in the Frankfurt plane. Body mass was obtained using the integrated scale of a multifrequency bioelectrical impedance analyzer (InBody 230, Biospace Co., Ltd., Seoul, Republic of Korea). Participants were assessed barefoot while wearing light sports clothing after removing all metallic objects (e.g., watches, jewelry, belts, and mobile phones) that could interfere with the bioelectrical impedance measurements.

Anthropometric measurements were performed following standardized procedures consistent with the recommendations of the International Society for the Advancement of Kinanthropometry (ISAK) to ensure measurement reliability and reproducibility [26,27]. Body mass index (BMI) was calculated as body mass divided by height squared (kg·m^−2^).

#### 2.3.2. Isometric Quadriceps Strength Assessment

Isometric quadriceps strength was assessed using a digital hanging load cell (Mini Crane Scale, model OCS-M, Gromy Industry Co., Ltd., Hangzhou, China), with a maximum capacity of 300 kg, a minimum load of 2 kg, and a resolution of 0.1 kg. The device was secured with a non-elastic strap anchored to a rigid metal structure. Before each testing session, the device was visually inspected for proper operating condition and zeroed without load using the TARE/ZERO function. No formal external calibration was performed specifically for the present study. Participants were seated with the hip and knee flexed at 90°, maintaining an upright trunk position with the arms crossed over the chest to minimize compensatory movements. The load cell was attached to the distal leg using a non-elastic strap positioned immediately proximal to the malleoli at the distal third of the tibia [28,29].

Before data collection, participants completed one submaximal familiarization trial followed by three maximal voluntary isometric contractions (MVICs) for each lower limb. Each contraction was sustained for 5 s, with a 60-s recovery period between consecutive trials, following previously described procedures for belt-stabilized isometric strength testing of the knee extensors [30]. Testing was consistently initiated with the right lower limb, followed by the left. During each trial, standardized verbal encouragement (“Push as hard as possible”) together with a verbal countdown was provided by the evaluator to promote maximal effort. For each MVIC, the peak force value displayed by the device was recorded. The highest value obtained across the three trials was retained for each lower limb and subsequently used to calculate bilateral quadriceps strength, as described in the Section 2.4.

The testing setup was adapted from previously described belt-stabilized isometric knee-extension protocols [28,29,30,31], particularly regarding participant positioning, joint angles, stabilization, and maximal voluntary contraction procedures.

#### 2.3.3. Vertical Jump Performance

Vertical jump performance was assessed using the MyJump3^®^ smartphone application (Carlos Balsalobre-Fernández, Spain) installed on a Samsung Galaxy S24 smartphone. All jumps were recorded at 240 frames per second (fps), and flight time and jump height were analyzed offline by the same evaluator using the MyJump3 application. This application has demonstrated excellent validity and reliability for measuring vertical jump height compared with force platforms [32,33,34].

Participants completed three vertical jump tests a standardized order: Squat Jump (SJ), Countermovement Jump (CMJ), and Abalakov Jump (ABK). For the SJ, participants adopted a static position with the knees flexed to approximately 90°, maintained this position for 2 s to eliminate the contribution of the stretch-shortening cycle, and then performed a maximal vertical jump while keeping their hands on their hips. The CMJ was performed from an upright standing position using a rapid downward countermovement without pausing before take-off, with the hands remaining on the hips throughout the movement [35,36]. The ABK was performed using the same countermovement procedure as the CMJ; however, participants were allowed to perform a coordinated arm swing during the countermovement and take-off to maximize jump height [34].

Participants completed three maximal attempts for each jump test. A 60-s recovery period was provided between consecutive attempts, and 2 min of rest was allowed between jump tests to minimize the effects of fatigue. During each trial, standardized verbal encouragement (“Jump as high as possible”) was provided by the evaluator to promote maximal effort. For each jump test, the highest value obtained across the three attempts was retained for statistical analysis.

#### 2.3.4. Cardiorespiratory Fitness Testing

Cardiorespiratory exercise testing was conducted as part of the physical assessment procedures included in the broader institutional Collaborative Project ID4011. Cardiorespiratory exercise testing (CPET) was performed on a mercury^®^ motorized treadmill (h/p/cosmos sports & medical GmbH, Nussdorf-Traunstein, Germany) using a MetaLyzer^®^ 3B-R3 metabolic cart (CORTEX Biophysik GmbH, Leipzig, Germany) interfaced with MetaSoft Studio software, version 5.16.0. Before each assessment, the system was calibrated according to the manufacturer’s recommendations, including gas analyzer calibration using certified reference gases and flow calibration with a 3-L calibration syringe to ensure measurement accuracy. Respiratory gas exchange was measured continuously on a breath-by-breath basis throughout the test.

Participants performed a maximal incremental exercise test following the Bruce treadmill protocol. The assessment was terminated upon voluntary exhaustion, inability to maintain the required treadmill speed, or the appearance of any clinical indication requiring test termination according to established exercise testing guidelines. Breath-by-breath respiratory gas-exchange and cardiovascular responses were continuously recorded and processed using MetaSoft Studio (CORTEX Biophysik GmbH, Leipzig, Germany), applying the moving-average smoothing procedures implemented in the software. VO_2_peak was obtained from the software-generated CPET report and corresponded to the highest processed oxygen-uptake value achieved during the incremental exercise test. Because the original raw breath-by-breath files were not available for retrospective reprocessing, the values generated and recorded at the time of each assessment were retained for analysis. All tests were supervised by the same evaluator. Maximal or near-maximal effort was evaluated using the physiological responses recorded during the test, including peak heart rate and respiratory exchange ratio (RER), together with voluntary exhaustion and the participant’s ability to maintain the prescribed treadmill workload.

The variables extracted for statistical analysis included absolute VO_2_peak (L·min^−1^), relative VO_2_peak (mL·kg^−1^·min^−1^), predicted absolute VO_2_peak (L·min^−1^), peak heart rate (HRpeak), percentage of predicted peak heart rate, respiratory exchange ratio (RER), and the first ventilatory threshold (VT1). Heart rate was continuously monitored throughout the test using a Polar H10 heart rate sensor (Polar Electro Oy, Kempele, Finland). HRpeak was defined as the highest heart rate recorded during the exercise test. Age-predicted maximal heart rate was calculated by MetaSoft Studio using the equation HRmax = 200 − age. The percentage of predicted peak heart rate was calculated as (measured HRpeak/predicted HRmax) × 100. VT1 was obtained from the MetaSoft Studio CPET analysis and corresponded to the first ventilatory threshold identified from the ventilatory and gas-exchange responses during incremental exercise. The value reported in the original software-generated CPET report was retained for statistical analysis. Predicted absolute VO_2_peak values were automatically generated by MetaSoft Studio using the Wasserman weight-based prediction algorithm implemented in the software [37]. Testing procedures followed the recommendations of the American College of Sports Medicine (ACSM) and the American Thoracic Society/American College of Chest Physicians (ATS/ACCP) for standardized cardiopulmonary exercise testing.

### 2.4. Statistical Analysis

Statistical analyses were performed using R software, version 4.6.1 (R Foundation for Statistical Computing, Vienna, Austria), primarily using functions from the base and stats packages. Data completeness and potential entry errors were examined before analysis. Continuous variables were summarized as mean ± standard deviation (SD) when normally distributed or as median and interquartile range (IQR) when distributions deviated from normality. Categorical variables were expressed as absolute and relative frequencies. Normality was assessed using the Shapiro–Wilk test, complemented by visual inspection of histograms and quantile–quantile (Q–Q) plots.

The primary analysis aimed to characterize the anthropometric, neuromuscular, and cardiorespiratory profile of the participants. Descriptive statistics were calculated for age, height, body mass, body mass index, quadriceps isometric force, vertical jump performance (Squat Jump [SJ], Countermovement Jump [CMJ], and Abalakov Jump [ABK]), and cardiopulmonary exercise testing variables, including absolute VO_2_peak, predicted VO_2_peak, relative VO_2_peak, respiratory exchange ratio (RER), peak heart rate (HRpeak), percentage of predicted heart rate, and the first ventilatory threshold (VT1). Quadriceps force values recorded by the load cell in kilogram-force (kgf) were converted to newtons (N) by multiplying each trial by 9.80665. The highest converted value obtained across the three trials was retained for each lower limb. Bilateral quadriceps isometric strength corresponded to the mean value of the right and left lower limbs. Where appropriate, 95% confidence intervals (95% CI) were reported to indicate the precision of the estimates.

As secondary exploratory analyses, associations among anthropometric characteristics, quadriceps isometric force, vertical jump performance, and cardiorespiratory variables were examined. Pearson’s correlation coefficients were calculated for variables meeting assumptions of normality and linearity, whereas Spearman’s rank correlation coefficients were used when these assumptions were not satisfied. Correlation coefficients were reported together with exact *p*-values. For the correlation analysis, the Benjamini–Hochberg false discovery rate (FDR) correction was applied to the complete family of 45 bivariate correlation tests performed among the 10 study variables. The complete set of correlation coefficients, unadjusted *p*-values, and FDR-adjusted *p*-values for all 45 comparisons is provided in Supplementary Table S1. Adjusted *p*-values are reported to control the expected proportion of false-positive findings arising from these multiple comparisons. No FDR correction was applied to the normality tests or the multiple linear regression analysis.

An exploratory multiple linear regression analysis was performed to examine the contribution of selected neuromuscular and cardiorespiratory variables to Countermovement Jump (CMJ) height. CMJ was selected as the dependent variable because it is the most widely used and ecologically valid indicator of lower-limb neuromuscular performance in soccer players, reflecting the contribution of the stretch–shortening cycle during explosive movements. Mean bilateral quadriceps isometric force (N) and relative VO_2_peak (mL·kg^−1^·min^−1^) were entered as independent variables based on their physiological relevance to neuromuscular and aerobic performance in youth soccer players and were selected a priori to maintain a parsimonious model considering the available sample size [17,38]. Regression results were reported as unstandardized regression coefficients (B), standardized regression coefficients (β), 95% confidence intervals, exact *p*-values, the coefficient of determination (R^2^), and adjusted R^2^. To examine whether the association between absolute quadriceps strength and CMJ performance could be influenced by body size, an additional sensitivity analysis was performed including body mass together with bilateral quadriceps isometric strength and relative VO_2_peak as predictors of CMJ height. Body mass was selected as the body-size covariate because it showed the strongest association with absolute quadriceps strength in the correlation analysis. Given the sample size, no additional anthropometric covariates were simultaneously included to avoid unnecessary model complexity.

Regression assumptions were evaluated through graphical and numerical assessment of linearity, normality, and homoscedasticity of residuals. Multicollinearity was assessed using the variance inflation factor (VIF), whereas influential observations were examined using standardized residuals, leverage values, and Cook’s distance. Observations with Cook’s distance greater than 4/n were considered potentially influential and were excluded in a sensitivity analysis. Given the cross-sectional design and relatively small sample size, the correlation and regression analyses were considered exploratory and should be interpreted with caution because of the limited precision of the estimates. All statistical tests were two-tailed, and statistical significance was set at *p* < 0.05.

## 3. Results

### 3.1. Participant Characteristics

**Figure 1:**
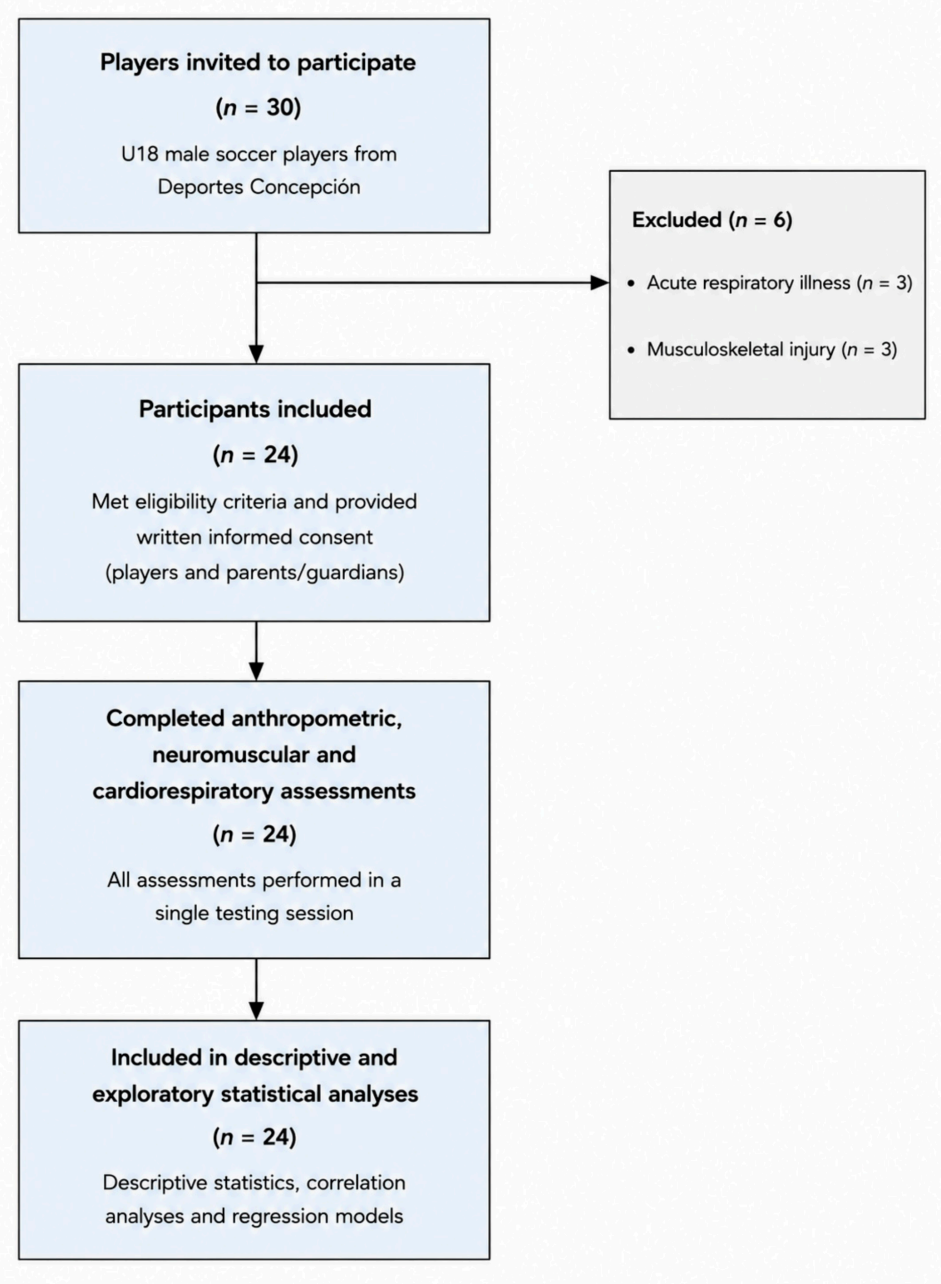
Flow diagram illustrating the recruitment process, eligibility screening, participant exclusions, completion of study assessments, and inclusion in the final statistical analysis. Thirty youth soccer players were invited to participate. Six players were excluded because of acute respiratory illness (*n* = 3) or musculoskeletal injury (*n* = 3). The remaining 24 participants completed all study assessments during a single testing session and were included in the descriptive, correlational, and regression analyses.

The participant flow throughout the study is presented in Figure 1. Of the 30 youth soccer players initially invited to participate, 24 completed all assessments and were included in the final analysis, whereas six players were excluded because of acute respiratory illness (*n* = 3) or musculoskeletal injury (*n* = 3). The descriptive characteristics of the included participants are presented in Table 1. Overall, the sample consisted of early adolescent male soccer players, predominantly right-leg dominant (83.3%), with forwards representing the largest positional group (45.8%).

The physical performance data are detailed in Table 2. Regarding neuromuscular performance, the average bilateral quadriceps strength is provided. The heights of the three jumps are reported in cm, showing progressively greater values from the Squat Jump (SJ) to the Countermovement Jump (CMJ) and Abalakov Jump (ABK). At the group level, the measured mean absolute VO_2_peak was slightly higher than the predicted value (3.27 vs. 3.23 L·min^−1^).

### 3.2. Exploratory Associations Between Anthropometric, Neuromuscular, and Cardiorespiratory Variables

Exploratory correlation analyses were performed to examine the associations among anthropometric, neuromuscular, and cardiorespiratory variables. The complete set of 45 correlations included in the FDR adjustment is presented in Supplementary Table S1, whereas Table 3 summarizes the associations most directly relevant to the study objectives. Height (*r* = 0.64, *p* < 0.001), body mass (*r* = 0.76, *p* < 0.001), and body mass index (*r* = 0.61, *p* = 0.001) were positively associated with bilateral quadriceps isometric strength. All three associations remained statistically significant after false discovery rate (FDR) adjustment. In contrast, neither age nor anthropometric variables showed significant associations with CMJ height.

**Figure 2:**
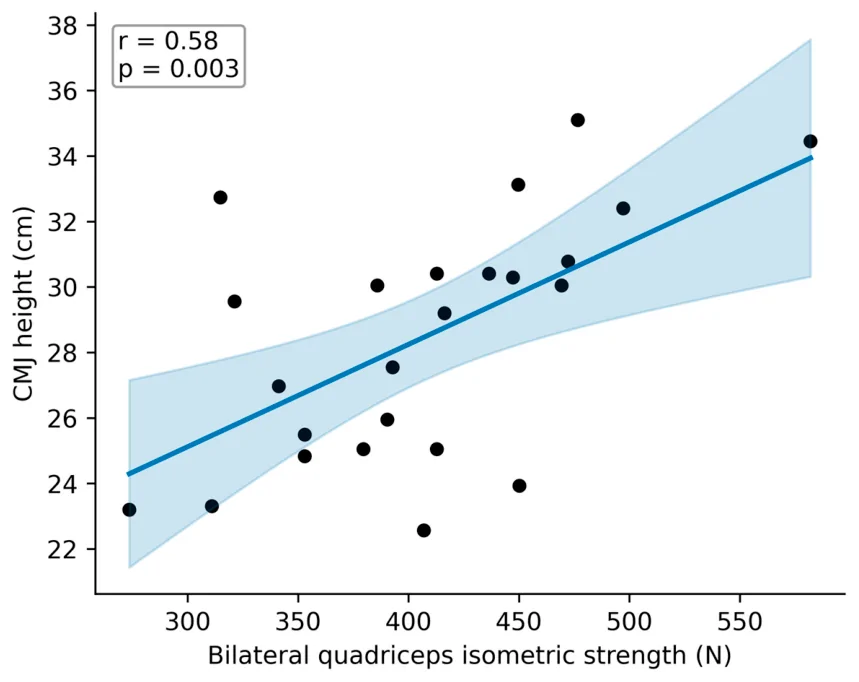
Association between bilateral quadriceps isometric strength and countermovement jump (CMJ) height. The solid blue line represents the fitted linear regression, and the shaded area indicates the 95% confidence interval.

Moderate positive associations were observed between bilateral quadriceps isometric strength and vertical jump performance. Specifically, quadriceps strength was positively associated with CMJ height (r = 0.58, *p* = 0.003; Figure 2), ABK height (r = 0.61, *p* = 0.002), and SJ height (r = 0.50, *p* = 0.013). After FDR adjustment, the associations with CMJ and ABK remained statistically significant, whereas the association with SJ did not (FDR-adjusted *p* = 0.055).

**Figure 3:**
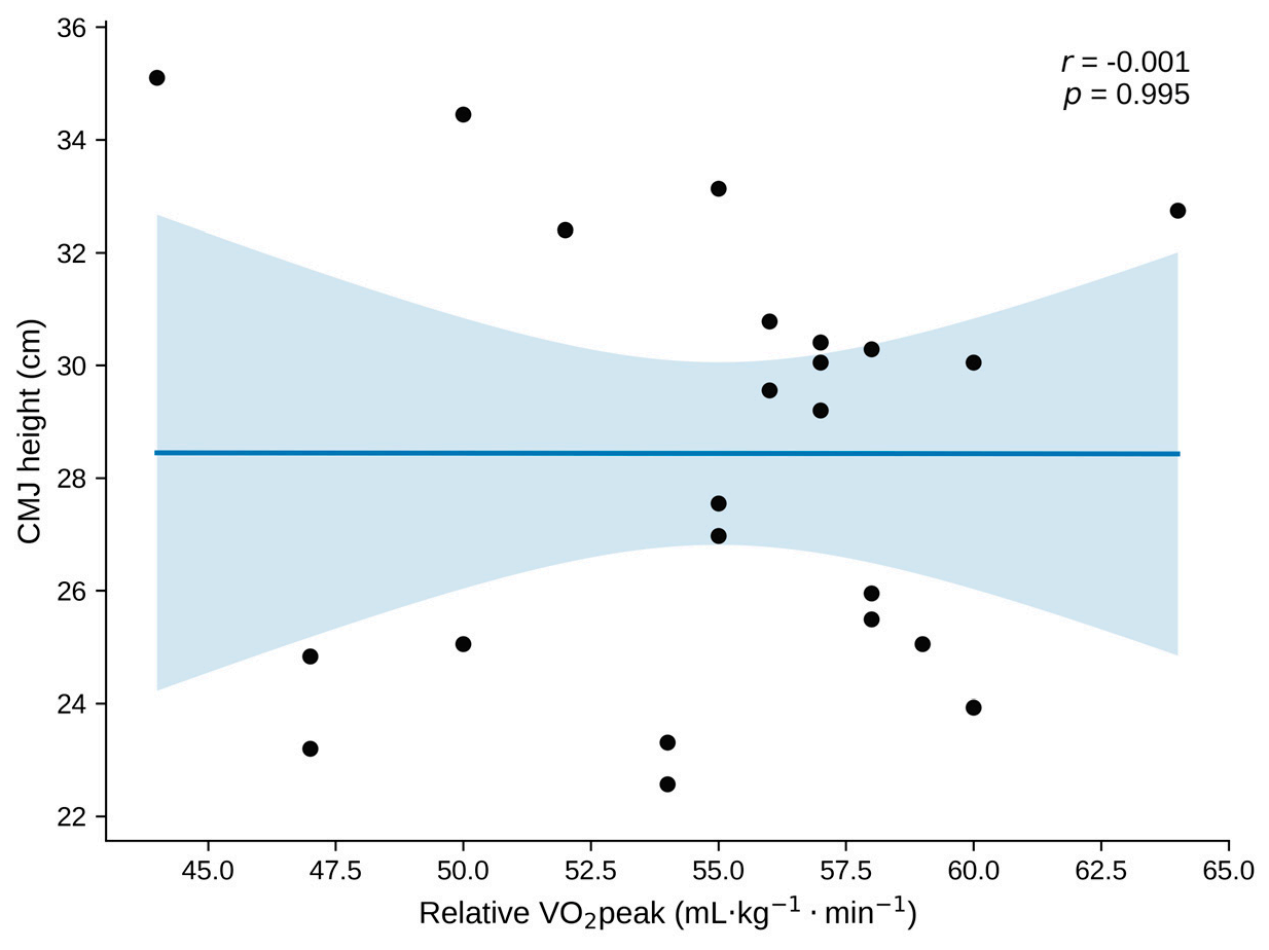
Association between relative peak oxygen uptake (VO_2_peak) and countermovement jump (CMJ) height. The solid blue line represents the fitted linear regression, and the shaded area indicates the 95% confidence interval.

In contrast, relative VO_2_peak was not significantly associated with CMJ performance (*r* = −0.001, *p* = 0.995; Figure 3). No other statistically significant associations were identified after adjustment for multiple comparisons.

### 3.3. Associations with Countermovement Jump Performance

Multiple linear regression analysis demonstrated that quadriceps isometric strength was associated with CMJ height after adjustment for relative VO_2_peak (Table 4). The overall model was statistically significant and explained 34% of the variance in CMJ performance (R^2^ = 0.34; adjusted R^2^ = 0.28; *p* = 0.012).

Quadriceps isometric strength was positively associated with CMJ height (standardized β = 0.59, *p* = 0.003), whereas relative VO_2_peak was not significantly associated with jump performance (standardized β = 0.04, *p* = 0.825).

To examine the potential influence of body size on this relationship, an additional sensitivity analysis was performed including body mass in the regression model. Bilateral quadriceps isometric strength remained significantly associated with CMJ height after adjustment for body mass and relative VO_2_peak (standardized β = 0.94, *p* = 0.002). In contrast, body mass (β = −0.46, *p* = 0.093) and relative VO_2_peak (β = 0.10, *p* = 0.584) were not significantly associated with CMJ height. The adjusted model explained 43.1% of the variance in CMJ performance (R^2^ = 0.431; adjusted R^2^ = 0.346; F(3,20) = 5.06, *p* = 0.009).

A sensitivity analysis was performed after excluding three observations identified as potentially influential using a Cook’s distance threshold of 4/n, resulting in a final sample of 21 participants. Quadriceps strength remained significantly associated with CMJ height (standardized β = 0.74, *p* < 0.001), whereas relative VO_2_peak remained non-significant (*p* = 0.541). The sensitivity model explained 58% of the variance in CMJ performance (R^2^ = 0.58; adjusted R^2^ = 0.53; F(2,18) = 12.48, *p* < 0.001). Given the increase in explained variance compared with the primary model, these findings suggest that individual observations influenced the magnitude of the estimated association and should therefore be interpreted cautiously.

## 4. Discussion

The present study characterized the anthropometric, neuromuscular, and cardiorespiratory fitness of a sample of competitive Chilean YSP, contributing descriptive evidence from a population that remains underrepresented in the scientific literature, despite the growing interest in youth soccer development in South America. The integrated assessment of anthropometric, neuromuscular, and cardiorespiratory performance provided a broader characterization of the physical profile of this cohort of competitive Chilean YSP than would have been obtained by evaluating these domains separately.

This study provides cohort-specific descriptive data on anthropometric characteristics, neuromuscular performance, and cardiorespiratory fitness in a sample of competitive Chilean YSP. Comparable data remain limited in South American populations despite potential differences in training environments, competitive structures, and developmental pathways relative to European cohorts. These findings may provide useful descriptive information for monitoring physical development, contextualizing players from similar competitive settings, and informing future longitudinal studies in South American youth soccer.

The anthropometric and physical performance characteristics observed in the present study were generally consistent with those reported in previous investigations involving YSP of similar age and competitive level [16,17,18,39]. Given the adolescent age range of the participants, BMI should be interpreted considering age- and sex-specific growth patterns rather than using adult BMI thresholds [40]. Similar values for body size, vertical jump performance, and aerobic capacity have been described in both Chilean and international cohorts, supporting the external consistency of the present findings. Nevertheless, these comparisons should be interpreted with caution, as between-study differences may partly reflect variations in biological maturation, training history, competitive level, and methodological aspects of testing protocols [3,11,17,21]. This highlights the importance of interpreting physical performance within the specific developmental and competitive context in which athletes are evaluated [41].

In regard to neuromuscular performance, quadriceps strength is one of the main determinants of force production during sprinting, jumping, and rapid changes of direction. The available evidence covers a wide range of assessment methods (squat, handgrip, isokinetic testing, among others), which makes it difficult to compare values. Nevertheless, despite these differences, the literature consistently reports greater quadriceps strength in older or higher-level soccer players, supporting the importance of knee extensor strength for soccer performance [42].

As expected, vertical jump performance increased progressively from the SJ to the CMJ and the ABK. This pattern is consistent with the biomechanical characteristics of these tests, as the greater jump height observed during the CMJ has been attributed to the contribution of the stretch–shortening cycle, whereas the additional increase during the ABK is explained by the mechanical contribution of arm swing, which enhances impulse generation during take-off [43]. Similar jump patterns have been reported in youth soccer players, indicating that the present findings are consistent with previous investigations in athletes of similar age and competitive level [10,36].

Cardiorespiratory fitness, commonly quantified as maximal oxygen uptake, is a key indicator of cardiovascular health in young populations [44]. The mean relative VO_2_peak observed in our sample (55.0 ± 4.7 mL·kg^−1^·min^−1^) is consistent with values reported in youth soccer players of comparable competitive level [45,46], although it remains below the 62–64 mL·kg^−1^·min^−1^ described in a large longitudinal analysis of professional soccer players evaluated over 23 years in Norway [47]. This difference may be due primarily to the fact that the study classifies U-20 players as “junior.” This category encompasses a wide age range with varying levels of development and biological maturity, which affect their performance metrics.

During adolescence, absolute VO_2_max tends to increase as cardiac output, hemoglobin concentration, and muscle mass rise. Moreover, early maturing adolescents usually exhibit higher absolute VO_2_max values than their late maturing peers of the same chronological age; however, these differences tend to diminish when oxygen consumption is expressed relative to body mass, underscoring the importance of appropriate scaling methods when interpreting cardiorespiratory fitness at this stage [23]. In line with this, relative VO_2_peak (mL·kg^−1^·min^−1^) obtained from an incremental laboratory test provides a more objective and robust measure of cardiorespiratory fitness than indirect field-based estimates, reinforcing the value of direct assessment as a monitoring tool in youth soccer [44].

One of the main findings of this study was the positive association between bilateral quadriceps isometric strength and vertical jump performance. Greater quadriceps strength was associated with higher SJ, CMJ, and ABK heights in the unadjusted analyses; however, after FDR correction, only the associations with CMJ and ABK remained statistically significant. Quadriceps strength remained significantly associated with CMJ height after adjustment for relative VO_2_peak and body mass in the sensitivity analysis. This finding suggests that the observed strength–CMJ association was not solely attributable to differences in body mass within this cohort. This finding agrees with previous studies identifying lower-limb strength as an important determinant of explosive performance in youth soccer players [19,21,48]. Keiner et al. [49] reported that greater maximal squat strength was associated with superior jumping and sprinting performance, whereas Martínez et al. [19] observed better squat jump performance in players with greater fat-free mass, indirectly supporting the contribution of muscular capacity to explosive actions. Although different methods were used to assess strength, these studies consistently suggest that greater lower-limb strength is associated with improved jumping performance. However, this relationship should be interpreted within the context of adolescent development. Perroni et al. [21] demonstrated that biological maturation substantially influences neuromuscular performance, while Diker et al. [48] showed that the strength–jump relationship varies according to the assessment method. Therefore, the present findings are likely explained by the combined influence of muscular strength, biological maturation, and neuromuscular development rather than by quadriceps strength alone.

In contrast, neither anthropometric characteristics nor relative VO_2_peak were associated with CMJ performance. This finding differs from previous studies reporting better physical performance in taller or heavier youth soccer players [16,17,18]. However, these differences are more likely attributable to variations in study populations than to conflicting physiological mechanisms. Most previous investigations included athletes spanning wider age ranges or different stages of biological maturation, where increases in stature, body mass, and lean mass occur concurrently with improvements in neuromuscular performance [3,11,18]. Under these circumstances, anthropometric characteristics may reflect biological maturation rather than independently influence explosive performance. By comparison, the participants in the present study represented a relatively homogeneous cohort with similar age, competitive level, and training exposure, reducing variability in body size. This may explain why anthropometric measures were not associated with CMJ performance, whereas neuromuscular characteristics appeared to better distinguish differences in explosive capacity [21,22].

Relative VO_2_peak was also not associated with CMJ performance, suggesting that aerobic fitness did not explain differences in explosive capacity within this cohort. This finding is consistent with the distinct physiological adaptations underlying aerobic and neuromuscular performance, which may develop independently during adolescence [11,20]. The relatively homogeneous age, competitive level, and training background of the participants may have further reduced variability in aerobic fitness, limiting its contribution to differences in jumping performance. These results indicate that higher aerobic capacity should not be interpreted as an indicator of superior explosive performance in youth soccer players. Instead, aerobic fitness and neuromuscular performance appear to provide complementary information and should therefore be evaluated independently when monitoring physical development and training adaptations [3].

The present findings contribute to the existing body of evidence and may provide useful descriptive information for the assessment of physical performance among Chilean YSP, while also supporting the use of multidimensional assessment strategies during the physical evaluation of youth soccer players [50,51]. Chilean YSP exhibit anthropometric characteristics and physical abilities comparable to those of similar populations, with some areas showing opportunities for improvement. Although CMJ is widely used to monitor neuromuscular performance, the present results indicate that it should not be interpreted as a surrogate measure of overall physical fitness. Likewise, greater aerobic capacity or more favorable anthropometric characteristics should not be assumed to indicate superior explosive performance in players of similar age and competitive level. These findings reinforce the importance of interpreting each physical domain according to the specific performance attribute it represents when monitoring youth soccer players.

This study has several strengths that should be considered when interpreting the findings. It provides an integrated characterization of anthropometric, neuromuscular, and cardiorespiratory fitness in competitive Chilean youth soccer players using standardized assessment procedures. Furthermore, the integration of anthropometric, neuromuscular, and cardiorespiratory assessments within a standardized laboratory setting enabled a comprehensive evaluation of physical performance within a single testing session. Nevertheless, several limitations should be acknowledged. First, the cross-sectional design precludes conclusions regarding causality, and the relatively small sample recruited from a single competitive club limits the generalizability of the findings and the precision of the exploratory correlation and regression estimates. Second, biological maturation was not assessed despite its well-established influence on body size, muscle strength, and neuromuscular performance during adolescence. Although the additional sensitivity analysis accounted for body mass, residual confounding related to growth and maturation cannot be excluded. Future studies should incorporate objective measures of biological maturation to better disentangle the contributions of growth, body size, and neuromuscular development to strength and jumping performance. In addition, quadriceps strength was assessed using a load-cell system without formal external calibration or device-specific validation; therefore, the absolute force values should be interpreted with appropriate caution. Finally, quadriceps strength was assessed under isometric conditions, whereas jumping performance is a dynamic multi-joint task. Therefore, the observed associations should not be interpreted as evidence that isometric strength alone determines explosive performance. Future longitudinal multicenter studies incorporating objective measures of biological maturation and dynamic strength assessments are warranted to clarify how growth, neuromuscular development, and physical performance interact throughout adolescence.

## 5. Conclusions

This study highlights the complementary value of multidimensional physical assessment in competitive Chilean youth soccer players. Within this cohort, neuromuscular performance, particularly quadriceps isometric strength, was more closely related to vertical jump performance than cardiorespiratory fitness, suggesting that these physical domains provide distinct rather than interchangeable information about athletic performance. Accordingly, the combined assessment of neuromuscular and cardiorespiratory characteristics may provide a more comprehensive understanding of individual physical profiles in youth soccer. Given the relatively small sample from a single club and the cross-sectional design, these findings should be interpreted as cohort-specific and exploratory. Larger, multicenter longitudinal studies incorporating biological maturation are needed to determine how these relationships evolve throughout player development.

## Figures and Tables

**Table 1:** Anthropometric characteristics and physical performance of the study participants (*n* = 24).

Variable	Value
Anthropometric characteristics	
Age (years)	13.5 (13.0–14.0)
Height (cm)	166.6 ± 7.0
Body mass (kg)	58.8 ± 7.4
Body mass index (kg·m^−2^)	21.1 ± 1.6
Soccer-related characteristics	
Right-leg dominance, *n* (%)	20 (83.3)
Time in current club, *n* (%)	
1 year	15 (62.5)
>1 year	9 (37.5)
Playing position, *n* (%)	
Defender	6 (25.0)
Midfielder	7 (29.2)
Forward	11 (45.8)

Data are presented as mean ± standard deviation (SD), median (interquartile range [IQR]), or number (percentage), as appropriate.

**Table 2:** Neuromuscular performance and cardiorespiratory fitness characteristics of the study participants (*n* = 24).

Variable	Value
Neuromuscular performance	
Bilateral quadriceps isometric strength (N)	406.1 ± 70.0
Squat jump height (cm)	24.6 ± 4.2
Countermovement jump height (cm)	28.4 ± 3.7
Abalakov jump height (cm)	33.4 ± 5.3
Cardiorespiratory fitness	
Absolute VO_2_peak (L/min)	3.27 ± 0.42
Predicted VO_2_peak (L/min)	3.23 ± 0.31
Relative VO_2_peak (mL·kg^−1^·min^−1^)	55.0 ± 4.7
Respiratory exchange ratio (RER)	1.12 ± 0.03
Peak heart rate (beats/min)	196 [192–199]
Percentage of predicted peak heart rate (%)	105.5 [102.8–107.0]
First ventilatory threshold (VT1) (L/min)	1.55 [1.40–1.81]

Data are presented as mean ± standard deviation (SD) for normally distributed variables and median [interquartile range (IQR)] for non-normally distributed variables. Bilateral quadriceps isometric strength represents the average force generated by both lower limbs. VO_2_peak = peak oxygen uptake; RER = respiratory exchange ratio; VT1 = first ventilatory threshold.

**Table 3:** Selected exploratory associations among anthropometric, neuromuscular, and cardiorespiratory variables.

Variables	Coefficient (r or ρ)	*p*-Value	FDR-Adjusted *p*-Value
Anthropometric variables and quadriceps strength			
Height vs. Bilateral quadriceps strength	0.64	<0.001	0.005
Body mass vs. Bilateral quadriceps strength	0.76	<0.001	<0.001
Body mass index vs. Bilateral quadriceps strength	0.61	0.001	0.008
Age vs. Bilateral quadriceps strength	0.29	0.163	0.386
Anthropometric variables and CMJ			
Height vs. CMJ	0.33	0.111	0.311
Body mass vs. CMJ	0.26	0.226	0.461
Body mass index vs. CMJ	0.07	0.729	0.861
Age vs. CMJ	0.10	0.652	0.861
Cardiorespiratory performance			
Relative VO_2_peak vs. CMJ	−0.001	0.995	0.995
Neuromuscular performance			
Bilateral quadriceps strength vs. SJ	0.50	0.013	0.055
Bilateral quadriceps strength vs. CMJ	0.58	0.003	0.012
Bilateral quadriceps strength vs. ABK	0.61	0.002	0.009

Pearson’s correlation coefficient (r) was used for normally distributed variables, whereas Spearman’s rank correlation coefficient (ρ) was used for age because of its non-normal distribution. False discovery rate (FDR) adjustment was performed using the Benjamini–Hochberg procedure. Statistically significant associations after FDR correction are shown in bold. The complete correlation analysis, including all 45 comparisons used for the FDR adjustment, is provided in Supplementary Table S1.

**Table 4:** Multiple linear regression analysis of bilateral quadriceps strength and relative VO_2_peak in relation to countermovement jump height.

Predictor	B	95% CI	Standardized β	SE	t	*p*-Value
Bilateral quadriceps isometric strength (N)	0.031	0.012 to 0.051	0.587	0.009	3.31	0.003
Relative VO_2_peak (mL·kg^−1^·min^−1^)	0.032	−0.264 to 0.327	0.040	0.142	0.22	0.825

Abbreviations: B, unstandardized regression coefficient; β, standardized regression coefficient; CI, confidence interval; SE, standard error; VO_2_peak, peak oxygen uptake. Model statistics: *n* = 24; R^2^ = 0.343; adjusted R^2^ = 0.281; F(2,21) = 5.48; overall model *p* = 0.012.

## Data Availability

The data presented in this study are available on request from the corresponding author. The data are not publicly available due to privacy and ethical restrictions related to participant confidentiality.

## Supplementary Materials

The following supporting information can be downloaded at: https://www.mdpi.com/article/10.3390/jfmk11030363/s1, Table S1. Complete exploratory bivariate correlation analysis among study variables.

## Abbreviations

The following abbreviations are used in this manuscript:

ABK	Abalakov Jump
ACCP	American College of Chest Physicians
ACSM	American College of Sports Medicine
ATS	American Thoracic Society
BMI	Body Mass Index
CI	Confidence Interval
CMJ	Countermovement Jump
CPET	Cardiopulmonary Exercise Testing
FDR	False Discovery Rate
HRpeak	Peak Heart Rate
IQR	Interquartile Range
ISAK	International Society for the Advancement of Kinanthropometry
MVIC	Maximal Voluntary Isometric Contraction
Q–Q	Quantile–Quantile
RER	Respiratory Exchange Ratio
SD	Standard Deviation
SE	Standard Error
SJ	Squat Jump
STROBE	Strengthening the Reporting of Observational Studies in Epidemiology
VIF	Variance Inflation Factor
VO_2_peak	Peak Oxygen Uptake
VT1	First Ventilatory Threshold
WHO	World Health Organization
YSP	Youth soccer players
