# Supplementary material for: Anthropometric Characteristics, Neuromuscular Performance and Cardiorespiratory Fitness of Chilean Youth Soccer Players: A Cross-Sectional Study
Source: J Funct Morphol Kinesiol. 2026 Sep 9;11(3):363. doi: 10.3390/jfmk11030363 (PMC13608819; doi:10.3390/jfmk11030363)
Supplement: Supplementary file 1 [file jfmk-11-00363-s001.zip › jfmk-4506092-supplementary.pdf]

Table S1. **Complete exploratory bivariate correlation analysis among study variables.**

The table reports all 45 bivariate correlations included in the Benjamini–Hochberg false discovery rate (FDR) adjustment (n = 24).

Variable 1	Variable 2	Coefficient (r or ρ)	p-value	FDR-adjusted p-value
**Age (years)**	Height (cm)	0.210	0.325	0.610
**Age (years)**	Body mass (kg)	0.215	0.313	0.610
**Age (years)**	Body mass index ($kg{\cdot}m^{-2}$)	0.150	0.486	0.705
**Age (years)**	Bilateral quadriceps isometric strength (N)	0.294	0.163	0.386
**Age (years)**	Squat jump height (cm)	0.343	0.101	0.302
**Age (years)**	Countermovement jump height (cm)	0.097	0.652	0.861
**Age (years)**	Abalakov jump height (cm)	0.204	0.340	0.612
**Age (years)**	Relative VO_2peak ($mL{\cdot}kg^{-1}{\cdot}min^{-1}$)	0.171	0.425	0.683
**Age (years)**	Respiratory exchange ratio (RER)	0.183	0.392	0.653
**Height (cm)**	Body mass (kg)	0.836	<0.001	<0.001
**Height (cm)**	Body mass index ($kg{\cdot}m^{-2}$)	0.351	0.093	0.299
**Height (cm)**	Bilateral quadriceps isometric strength (N)	0.636	<0.001	0.005
**Height (cm)**	Squat jump height (cm)	0.299	0.156	0.386
**Height (cm)**	Countermovement jump height (cm)	0.334	0.111	0.311
**Height (cm)**	Abalakov jump height (cm)	0.469	0.021	0.078
**Height (cm)**	Relative VO_2peak ($mL{\cdot}kg^{-1}{\cdot}min^{-1}$)	0.002	0.992	0.995
**Height (cm)**	Respiratory exchange ratio (RER)	-0.152	0.479	0.705
**Body mass (kg)**	Body mass index ($kg{\cdot}m^{-2}$)	0.804	<0.001	<0.001
**Body mass (kg)**	Bilateral quadriceps isometric strength (N)	0.758	<0.001	<0.001
**Body mass (kg)**	Squat jump height (cm)	0.288	0.172	0.388
**Body mass (kg)**	Countermovement jump height (cm)	0.257	0.226	0.461
**Body mass (kg)**	Abalakov jump height (cm)	0.452	0.027	0.093
**Body mass (kg)**	Relative VO_2peak ($mL{\cdot}kg^{-1}{\cdot}min^{-1}$)	0.068	0.753	0.861
**Body mass (kg)**	Respiratory exchange ratio (RER)	0.071	0.741	0.861
**Body mass index ($kg{\cdot}m^{-2}$)**	Bilateral quadriceps isometric strength (N)	0.614	0.001	0.008
**Body mass index ($kg{\cdot}m^{-2}$)**	Squat jump height (cm)	0.196	0.360	0.623
**Body mass index ($kg{\cdot}m^{-2}$)**	Countermovement jump height (cm)	0.075	0.729	0.861
**Body mass index ($kg{\cdot}m^{-2}$)**	Abalakov jump height (cm)	0.282	0.181	0.388
**Body mass index ($kg{\cdot}m^{-2}$)**	Relative VO_2peak ($mL{\cdot}kg^{-1}{\cdot}min^{-1}$)	0.117	0.588	0.801
**Body mass index ($kg{\cdot}m^{-2}$)**	Respiratory exchange ratio (RER)	0.316	0.132	0.350
**Bilateral quadriceps isometric strength (N)**	Squat jump height (cm)	0.498	0.013	0.055
**Bilateral quadriceps isometric strength (N)**	Countermovement jump height (cm)	0.584	0.003	0.012
**Bilateral quadriceps isometric strength (N)**	Abalakov jump height (cm)	0.606	0.002	0.009
**Bilateral quadriceps isometric strength (N)**	Relative VO_2peak ($mL{\cdot}kg^{-1}{\cdot}min^{-1}$)	-0.070	0.747	0.861
**Bilateral quadriceps isometric strength (N)**	Respiratory exchange ratio (RER)	0.044	0.838	0.884
**Squat jump height (cm)**	Countermovement jump height (cm)	0.696	<0.001	0.001

**Squat jump height (cm)**	Abalakov jump height (cm)	0.785	<0.001	<0.001
**Squat jump height (cm)**	Relative VO_2peak ($mL \cdot kg^{-1} \cdot min^{-1}$)	0.059	0.784	0.861
**Squat jump height (cm)**	Respiratory exchange ratio (RER)	0.086	0.690	0.861
**Countermovement jump height (cm)**	Abalakov jump height (cm)	0.666	<0.001	0.003
**Countermovement jump height (cm)**	Relative VO_2peak ($mL \cdot kg^{-1} \cdot min^{-1}$)	-0.001	0.995	0.995
**Countermovement jump height (cm)**	Respiratory exchange ratio (RER)	0.042	0.845	0.884
**Abalakov jump height (cm)**	Relative VO_2peak ($mL \cdot kg^{-1} \cdot min^{-1}$)	-0.163	0.447	0.693
**Abalakov jump height (cm)**	Respiratory exchange ratio (RER)	0.123	0.567	0.797
**Relative VO_2peak ($mL \cdot kg^{-1} \cdot min^{-1}$)**	Respiratory exchange ratio (RER)	0.064	0.765	0.861

Notes: Pearson's correlation coefficient (r) was used for normally distributed variables. Spearman's rank correlation coefficient (ϱ) was used for correlations involving age because age deviated from normality. FDR-adjusted p-values were calculated across the complete family of 45 bivariate tests using the Benjamini–Hochberg procedure. Statistical significance was set at $p < 0.05$. VO_2peak, peak oxygen uptake.
